# Supplementary material for: Potent induction of humoral and cellular immunity after bivalent BA.4/5 mRNA vaccination in dialysis patients
Source: NPJ Vaccines. 2024 Feb 7;9:25. doi: 10.1038/s41541-024-00816-0 (PMC10850212; doi:10.1038/s41541-024-00816-0)
Supplement: Supplementary file 1 — Supplementary material [file 41541_2024_816_MOESM1_ESM.docx]

**Supplementary material**

**Potent induction of humoral and cellular immunity after bivalent BA.4/5 mRNA vaccination in dialysis patients**

Saskia Bronder, MSc^1^, Janine Mihm, MD^2^, Rebecca Urschel, MSc^1^, Verena Klemis, Dipl. Biol.^1^, Tina Schmidt, PhD^1^, Stefanie Marx, Dipl. Biol.^1^, Amina Abu-Omar^1^, Franziska Hielscher, MSc^1^, Candida Guckelmus^1^, Marek Widera, PhD^3^, Urban Sester, MD^2^, and Martina Sester, PhD^1;4*^

**Table of content**

[Supplementary Figures 1](#_Toc1337890625)

[Supplementary Figure 1 2](#_Toc1634328015)

[Supplementary Figure 2 2](#_Toc1741557622)

[Supplementary Figure 3 4](#_Toc1100653212)

[Supplementary Figure 4 6](#_Toc1731843467)

[Supplementary Figure 5 8](#_Toc947519665)

[Supplementary Tables 10](#_Toc54087735)

[Supplementary Table 1: Results of multivariate regression analyses 11](#_Toc2014368700)

[Supplementary Table 2. Demographic and clinical characteristics of patients and healthy non-dialysing controls. 11](#_Toc185550162)

# Supplementary Figures

## Supplementary Figure 1


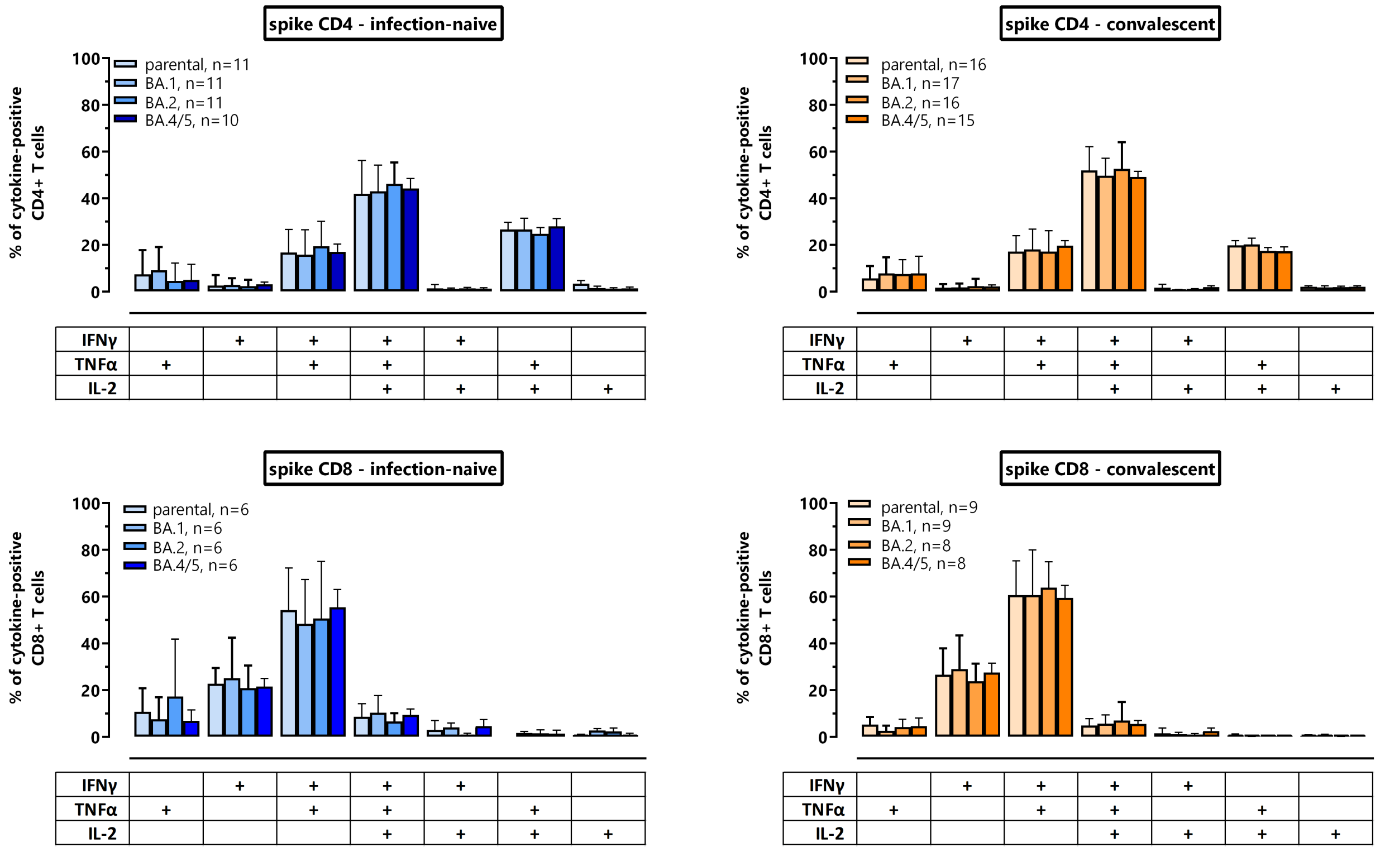


**Supplementary Figure 1: Cytokine expression profiles of CD4^+^ and CD8^+^ T cells specific for parental spike and spike of Omicron subvariants.** Comparison of cytokine-expression profile of CD4^+^ and CD8^+^ T cells after respective stimulation with parental spike as well as the Omicron variants BA.1-, BA.2 and BA.4/5-spike peptides in patients without and with prior infection. Cytokine-expressing T-cells were differentiated into 7 subpopulations according to their expression of IFNγ, TNFα and IL-2 (single, double or triple cytokine-expressing cells). Only samples with at least 30 cytokine-expressing CD4^+^ and CD8^+^ T cells were included, respectively, to ensure robust statistical analysis. Bars represent means and standard deviations. Differences among subpopulations between the groups were determined using Kruskal-Wallis test with Dunn's multiple comparisons post test. There were no significant differences between the cytokine profiles.

## Supplementary Figure 2


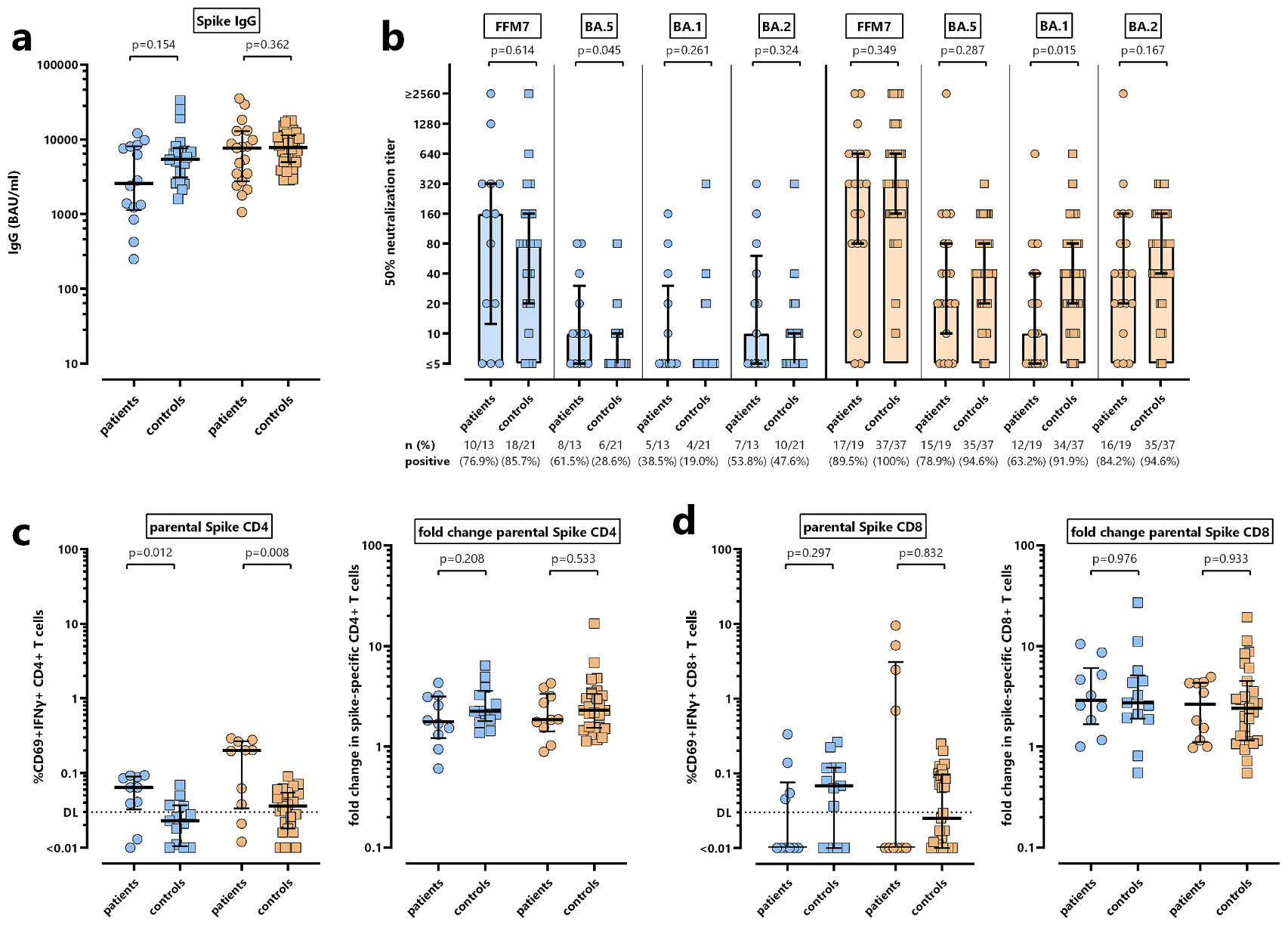


**Supplementary Figure 2: Comparison of SARS-CoV-2 specific humoral and cellular immunity between dialysis patients and healthy controls prior to bivalent vaccination.** **(a)** Spike-specific IgG levels prior to bivalent vaccination were compared between infection-naive dialysis patients (n=14, blue circle) and controls (n=21, blue square) as well as convalescent dialysis patients (n=19, orange dots) and controls (n=37; orange squares). **(b)** Antibody-mediated neutralization of parental SARS-CoV-2 strain (FFM7) and Omicron BA.1, BA.2 and BA.5 variants of concern before vaccination with the bivalent vaccine (expressed as 50% neutralization titers) was compared between dialysis patients and controls with and without prior infection. The number of tested individuals and the percentage of individuals with detectable neutralizing antibodies are indicated. Pre-vaccination levels and fold change of **(c)** CD4 and **(d)** CD8 T cells towards spike from the parental strain were compared in patients and controls with and without prior infection. Pre-vaccination tests were available from 10 patients each with and without prior infection, and from 27 controls with and 13 controls without prior infection. Dotted lines indicate detection limits (DL) for spike-specific CD4^+^ and CD8^+^ T cells. Fold changes after vaccination were calculated by dividing the individual levels after vaccination and levels prior to vaccination (with 0.03% added to each value prior to division to avoid division by 0). Bars represent median values with interquartile ranges. Differences between the groups were determined using Mann-Whitney test.

## Supplementary Figure 3


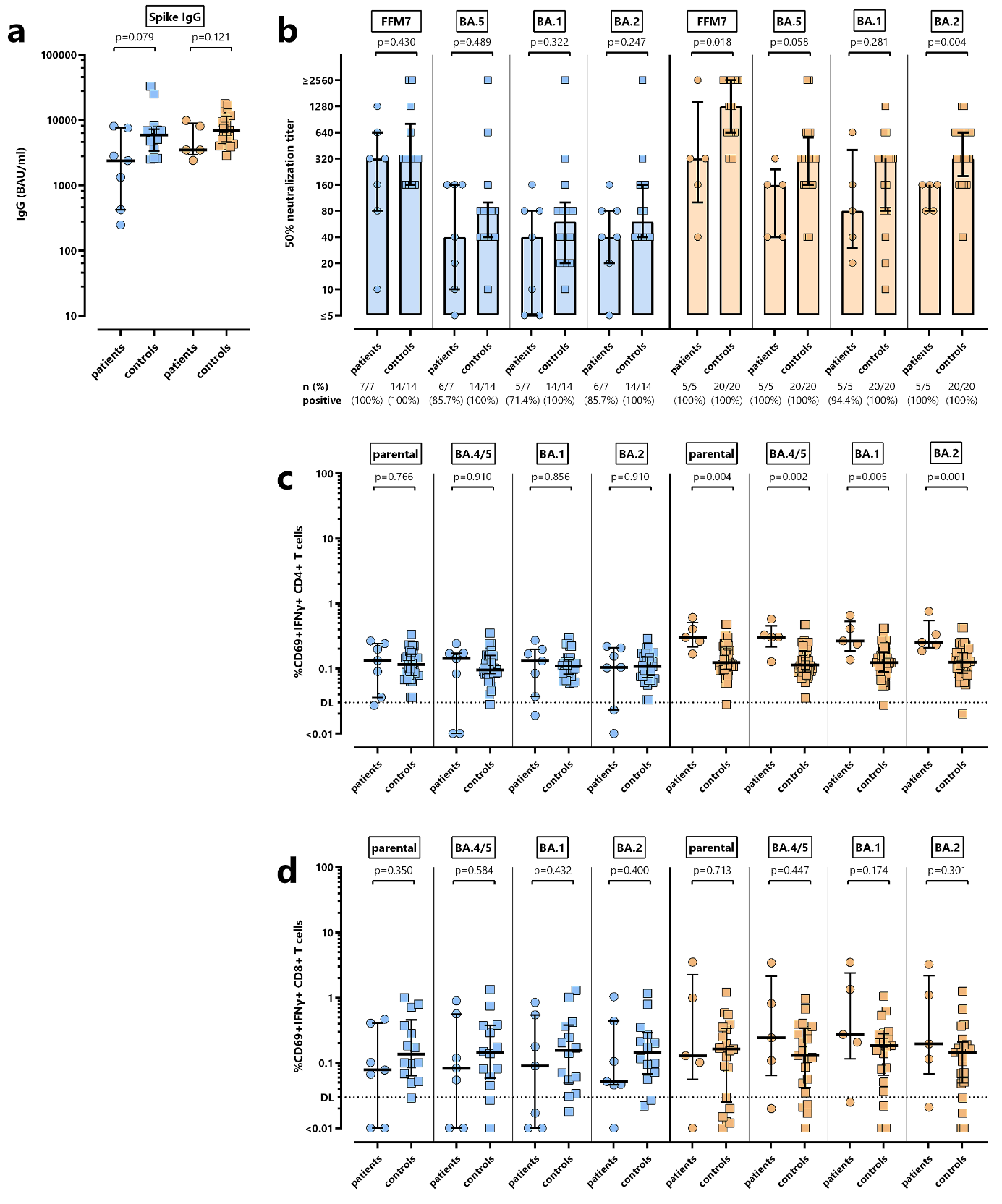


**Supplementary Figure 3: Disaggregated data for SARS-CoV-2 specific humoral and cellular immunity between female dialysis patients and female healthy controls prior to bivalent vaccination. (a)** Spike-specific IgG levels prior to bivalent vaccination were compared between infection-naive dialysis patients (n=7, blue circle) and controls (n=14, blue square) as well as convalescent dialysis patients (n=5, orange dots) and controls (n=21; orange squares). **(b)** Antibody-mediated neutralization of parental SARS-CoV-2 strain (FFM7) and Omicron BA.1, BA.2 and BA.5 variants of concern after vaccination with the bivalent vaccine (50% neutralization titers) was compared between dialysis patients and controls with and without prior infection. The number of tested individuals and the percentage of individuals with detectable neutralizing antibodies are indicated. Levels of (c) CD4 and (d) CD8 T cells towards spike from the parental strain and Omicron subvariants BA.4/5, BA.1, BA.2 after vaccination were compared in patients and controls with and without prior infection. Dotted lines indicate detection limits (DL) for spike-specific CD4+ and CD8+ T cells. Bars represent median titers with interquartile ranges. Differences between the groups were determined using Mann-Whitney test.

## Supplementary Figure 4


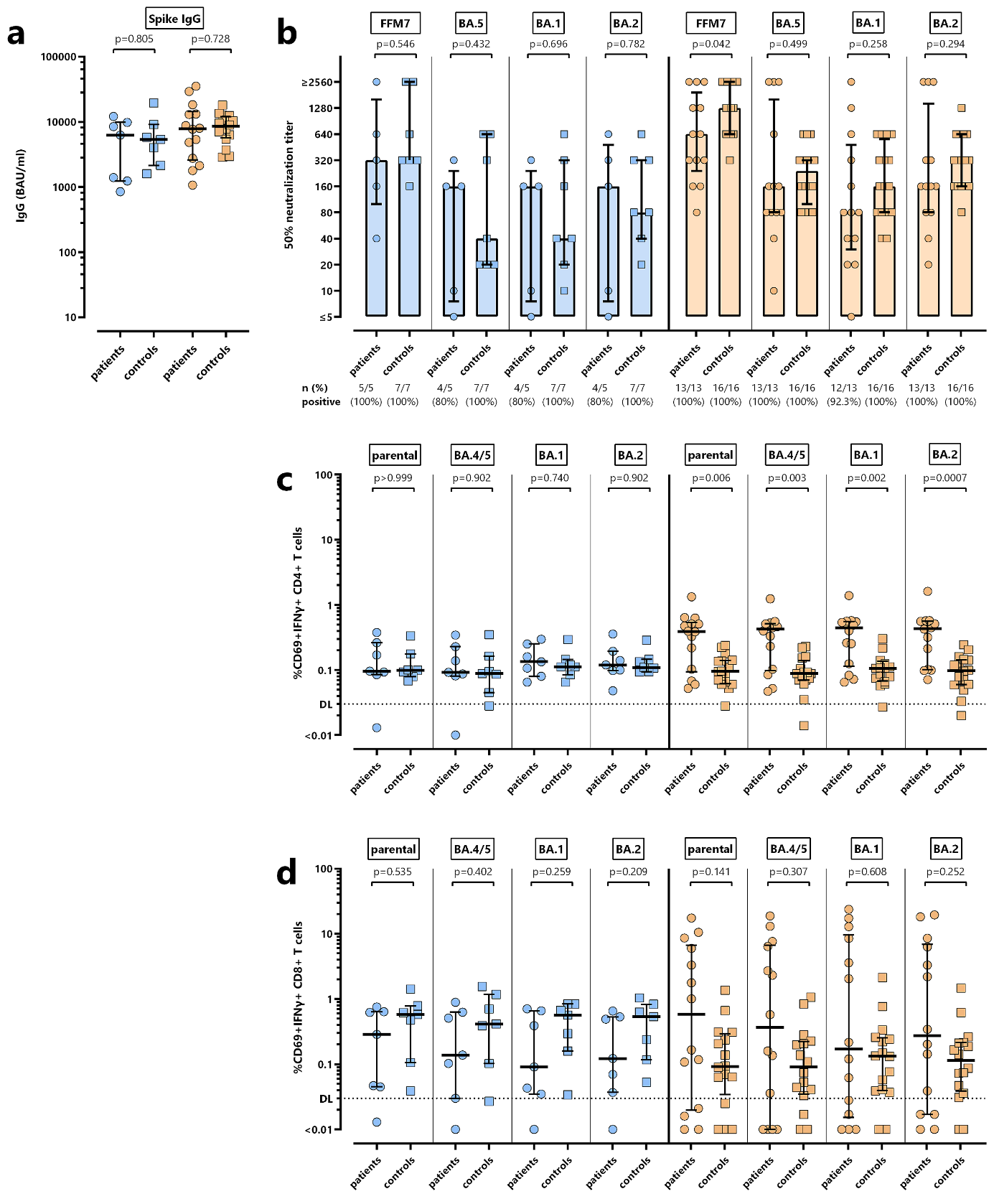


**Supplementary Figure 4: Disaggregated data for SARS-CoV-2 specific humoral and cellular immunity between male dialysis patients and male healthy controls prior to bivalent vaccination. (a)** Spike-specific IgG levels prior to bivalent vaccination were compared between infection-naive dialysis patients (n=7, blue circle) and controls (n=7, blue square) as well as convalescent dialysis patients (n=14, orange dots) and controls (n=16; orange squares). **(b)** Antibody-mediated neutralization of parental SARS-CoV-2 strain (FFM7) and Omicron BA.1, BA.2 and BA.5 variants of concern after vaccination with the bivalent vaccine (50% neutralization titers) was compared between dialysis patients and controls with and without prior infection. The number of tested individuals and the percentage of individuals with detectable neutralizing antibodies are indicated. Levels of (c) CD4 and (d) CD8 T cells towards spike from the parental strain and Omicron subvariants BA.4/5, BA.1, BA.2 after vaccination were compared in patients and controls with and without prior infection. Dotted lines indicate detection limits (DL) for spike-specific CD4+ and CD8+ T cells. Bars represent median titers with interquartile ranges. Differences between the groups were determined using Mann-Whitney test.

## Supplementary Figure 5


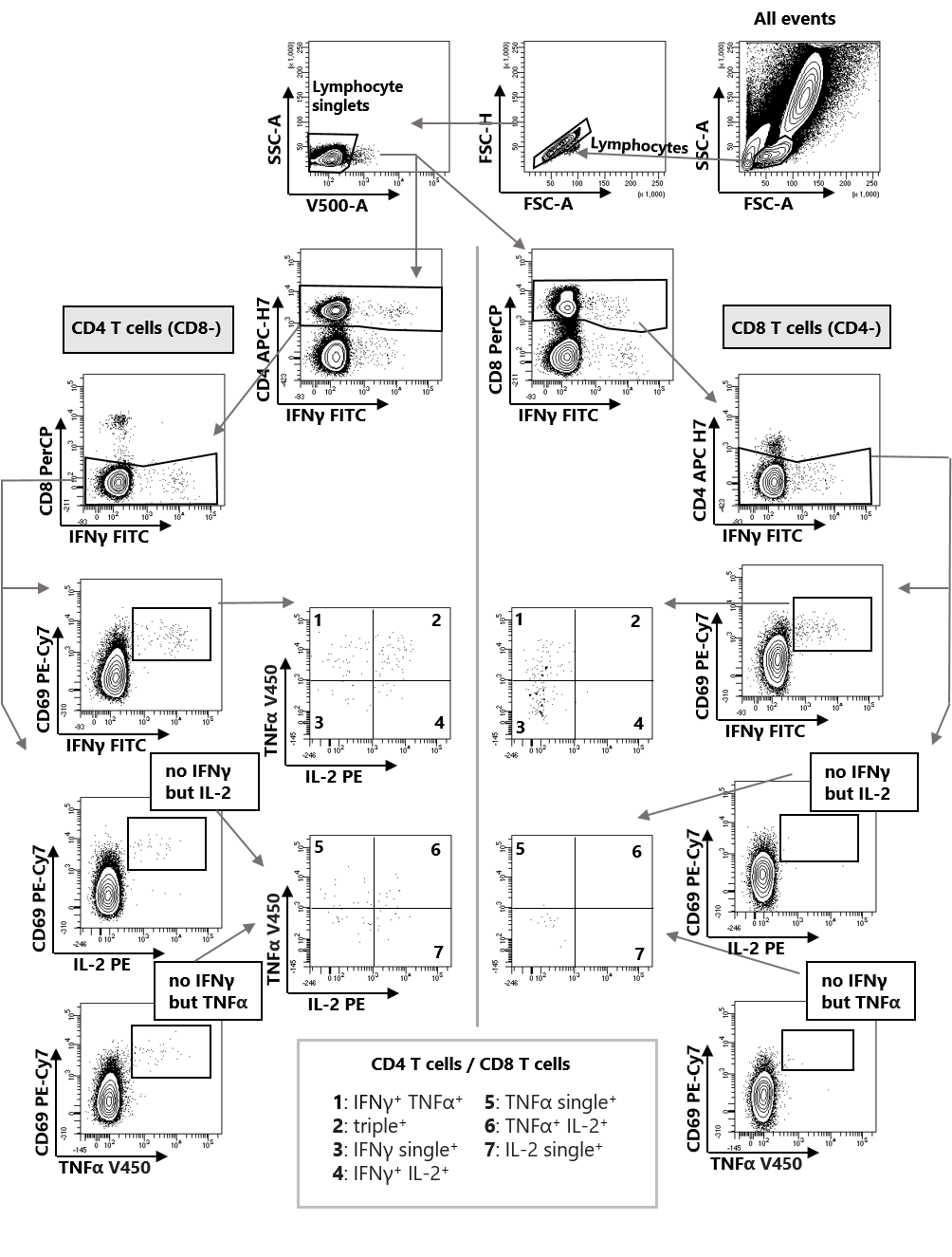


**Supplementary Figure 5: Gating strategy for identification of antigen-specific CD4 and CD8 T cells after stimulation.** Lymphocytes were identified among total events by backgating of CD4 and/or CD8 positive cells combined with signals for size (FSC) and granularity (SSC). Hight and area signals of FSC were used to exclude doublets. The gating strategy to identify CD4^+^ T cells (left side) or CD8^+^ T cells (right side) co-expressing the activation marker CD69 and the cytokines IFNγ, IL-2 or TNFα are shown. Boxes were used as gates to quantify the percentage of CD69^+^/IFNγ^+^ CD4^+^ or CD8^+^ T cells. Moreover, Boolean gating for cytokine profiling is shown that was used to identify subpopulations of CD4^+^ or CD8^+^ T cells expressing all three cytokines (triple^+^), two cytokines or one cytokine only. In table 1 and table S2, CD4^+^ or CD8^+^ T cells were calculated as percentage of lymphocytes from samples of the negative control stimulation.

# Supplementary Tables

## S**upplementary Table 1: Results of multivariate regression analyses**

| Confounders |  | Prior infection | Age | Number of previous immunization events |
| --- | --- | --- | --- | --- |
| Spike-specific IgG, parental | | **0.024** | 0.444 | 0.801 |
| Neutralizing antibodies | |  |  |  |
|  | FFM7 (parental) | 0.093 | 0.217 | 0.521 |
|  | BA.1 | 0.061 | 0.352 | 0.550 |
|  | BA.2 | **0.016** | 0.466 | 0.343 |
|  | BA.5 | **0.017** | 0.318 | 0.179 |
| Spike-specific CD4+ T cells | |  |  |  |
|  | parental | **0.0021** | 0.333 | 0.152 |
|  | BA.1 | **0.0004** | 0.074 | 0.355 |
|  | BA.2 | **0.0001** | 0.060 | 0.356 |
|  | BA.4/5 | **0.0016** | 0.352 | 0.436 |
| Spike-specific CD8+ T cells | |  |  |  |
|  | parental | 0.099 | 0.571 | 0.325 |
|  | BA.1 | 0.099 | 0.541 | 0.650 |
|  | BA.2 | 0.133 | 0.744 | 0.505 |
|  | BA.4/5 | 0.120 | 0.415 | 0.596 |

Shown are p-values of multiple linear regression analyses with log(10) transformed values.

## Supplementary Table 2. Demographic and clinical characteristics of patients and healthy non-dialysing controls.

|  |  | infection-naive | |  | convalescent | |  |
| --- | --- | --- | --- | --- | --- | --- | --- |
|  |  | dialysis patients | controls |  | dialysis patients | controls |  |
|  |  | n=14 | n=21 | p-value | n=19 | n=37 | p-value |
| Years of age, mean (SD) | | 71.8 (11.3) | 66.4 (6.6) | 0.082^1^ | 58.7 (15.5) | 55.9 (9.5) | 0.395^1^ |
| Sex, n (%)^4^ | | | | |  |  |  |
|  | Female | 7 (50.0%) | 14 (66.7%) | 0.296^2^ | 5 (26.3%) | 21 (56.8%) | 0.051^2^ |
|  | Male | 7 (50.0%) | 7 (33.3%) |  | 14 (73.7%) | 16 (43.2%) |  |
| Vaccine regimen, n (%) | |  |  |  |  |  |  |
|  | homologous mRNA | 13 (92.9%) | 12 (57.1%) |  | 17 (89.5%) | 25 (67.6%) |  |
|  | heterologous | 1 (7.1%) | 9 (42.9%) |  | 2 (10.5%) | 12 (32.4%) |  |
| Infecting strain^5^, n | |  |  |  |  |  |  |
|  | Parental SARS-CoV-2 | n.a. | n.a. |  | 12 | 4 |  |
|  | Delta | n.a. | n.a. |  | 1 | 1 |  |
|  | BA.1 | n.a. | n.a. |  | 2 | 5 |  |
|  | BA.2 | n.a. | n.a. |  | 4 | 21 |  |
|  | BA.4/5 | n.a. | n.a. |  | 0 | 4 |  |
|  | Unknown^6^ | n.a. | n.a. |  | 2 | 3 |  |
| Weeks between last infection and bivalent vaccination, median (IQR) | | n.a. | n.a. |  | 86.3 (57.6) | 31.1 (6.3) | 0.0006^3^ |
| Weeks between last immunization event and bivalent vaccination, median (IQR) | | 43.2 (23.2) | 45.0 (6.4) | 0.671^3^ | 40.0 (12.9) | 31.3 (7.5) | 0.031^3^ |
| Analysis time  (days after vaccination), median (IQR) | | 16 (2) | 15 (5) | 0.843^1^ | 16 (2) | 14 (2) | 0.183^1^ |
| Differential blood counts  median (IQR) cells/µl) | | n=13 | n=21 |  | n=19 | n=36 |  |
|  | Leukocytes | 6600 (4550) | 6900 (3000) | 0.511^3^ | 5800 (1900) | 7200 (2050) | 0.015^3^ |
|  | Granulocytes | 4891 (2536) | 3920 (2465) | 0.807^3^ | 4437 (1764) | 4267 (1878) | 0.424^3^ |
|  | Monocytes | 704 (266) | 662 (335) | 0.462^3^ | 536 (130) | 596 (298) | 0.249^3^ |
|  | Lymphocytes | 1280 (663) | 2134 (760) | 0.002^3^ | 1051 (480) | 2297 (700) | <0.0001^3^ |
| Percentage of T cells median (IQR) % among lymphocytes | |  |  |  |  |  |  |
|  | CD4 T cells | 36.0 (23.0) | 36.1 (7.7) | 0.908^3^ | 34.9 (11.6) | 41.4 (9.5) | 0.061^3^ |
|  | CD8 T cells | 18.4 (18.9) | 20.1 (13.6) | 0.454^3^ | 18.9 (9.8) | 18.3 (10.6) | 0.918^3^ |

^1^unpaired t-test, ^2^Fisher‘s exact test ^3^Mann-Whitney test; ^4^information on sex was based on individual self-declaration; ^5^infecting strain based on dominance of SARS-CoV-2 strain at the time of individual infection, 3 individuals had an infection with parental SARS-CoV-2 followed by a second infection with BA.1 (1 patient), BA.2 (1 patient) or BA.4/5 (1 control); ^6^individuals with no known history of infection with a positive NCAP-IgG.
